# Embedded metallic nanoparticles facilitate metastability of switchable metallic domains in Mott threshold switches

**DOI:** 10.1038/s41467-022-32081-x

**Published:** 2022-08-10

**Authors:** Minguk Jo, Ye-Won Seo, Hyojin Yoon, Yeon-Seo Nam, Si-Young Choi, Byung Joon Choi, Junwoo Son

**Affiliations:** 1 Department of Materials Science and Engineering (MSE), Pohang University of Science and Technology (POSTECH), Pohang, Republic of Korea; 2 Department of Chemical Engineering and Materials Science (CEMS), University of Minnesota, Minneapolis, MN USA; 3 Department of Materials Science and Engineering (MSE), Seoul National University of Science and Technology (Seoultech), Seoul, Republic of Korea

**Keywords:** Electronic properties and materials, Phase transitions and critical phenomena, Surfaces, interfaces and thin films

## Abstract

Mott threshold switching, which is observed in quantum materials featuring an electrically fired insulator-to-metal transition, calls for delicate control of the percolative dynamics of electrically switchable domains on a nanoscale. Here, we demonstrate that embedded metallic nanoparticles (NP) dramatically promote metastability of switchable metallic domains in single-crystal-like VO_2_ Mott switches. Using a model system of Pt-NP-VO_2_ single-crystal-like films, interestingly, the embedded Pt NPs provide 33.3 times longer ‘memory’ of previous threshold metallic conduction by serving as pre-formed ‘stepping-stones’ in the switchable VO_2_ matrix by consecutive electical pulse measurement; persistent memory of previous firing during the application of sub-threshold pulses was achieved on a six orders of magnitude longer timescale than the single-pulse recovery time of the insulating resistance in Pt-NP-VO_2_ Mott switches. This discovery offers a fundamental strategy to exploit the geometric evolution of switchable domains in electrically fired transition and potential applications for non-Boolean computing using quantum materials.

## Abstract (web summary)

Control of percolative dynamics of metal and insulator domains during electrically triggered insulator-metal transition underlies applications in energy-efficient switches. Jo et al. show that embedded metallic nanoparticles enhance the metastability and memory effects of metallic domains in VO_2_ switches.

## Introduction

Quantum materials featuring an abrupt metal-insulator transition have fascinated researchers for their variety of potential applications in future electronics^1–10^. Due to the extreme sensitivity of the electronic phase transition between competing phases, a subtle perturbation by external stimuli can abruptly transform an existing phase into a different electronic phase, leading to steep modulation of the electrical properties^7–12^. A characteristic phenomenon during the first order metal-insulator transition is the appearance of phase separation with metallic and insulating domains with inhomogeneous distributions down to a few nanometers^11,13–17^. The existence of phase separation implies that the resistance modulation occurs through a series of percolation transforming parts of the system from one phase to the other^2,11,13–19^. This percolative nature allows for an inhomogeneous transitional state where both metallic and insulating phases coexist; the dynamics of percolative domains in the intermediate state determines the macroscopic properties related to phase transition in quantum materials^2,11,13–19^.

Vanadium dioxide (VO_2_) undergoes a reversible transition between a monoclinic insulating phase and a rutile metallic phase near room temperature^13,20,21^. This thermally induced transition results in a giant modulation of electrical resistivity of up to five orders of magnitude, accompanied by changes in crystal symmetry and optical properties^7,9,13–15,20,22^. Uniformly distributed thermal energy over the whole VO_2_ results in a spatially random generation of nanoscale metallic puddles; these metallic puddles nucleate and then grow as metallic domains in the insulating matrix with increasing temperature, and eventually connect the entire area of VO_2_ as a result of gradual percolation^7,9,13–15,19,20,22^. The metallic domains are destabilized with decreasing temperature in a reversible manner.

In addition to temperature as an external stimulus, the insulator-to-metal transition (IMT) can be electrically stimulated on a subnanosecond time scale by applying an external voltage on two-terminal VO_2_ devices if a threshold voltage (*V*_*th*_) is exceeded^2,7,14,16–18,23–26^. A reverse metal-to-insulator transition (MIT) can promptly occur once the electrical stimulus is removed. These abrupt transitions by electrical stimuli have made VO_2_ a candidate for threshold switches in potential applications of low-voltage logic devices for energy-efficient switches^27^ and in artificial spiking neurons and synapses for non-Boolean computing^2,4,6^ to resolve the bottleneck in the state-of-the-art electronic devices.

**Fig. 1:**
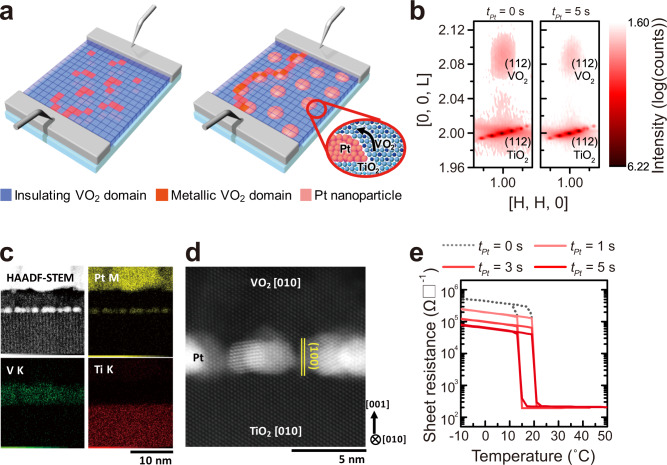
Single-crystal-like VO_2_ films embedded with Pt nanoparticle (NP). **a** Schematics of epitaxial VO_2_ films on (001)-TiO_2_ substrates and Pt NP-decorated (001)-TiO_2_ substrates. VO_2_ epitaxial films are selectively nucleated on TiO_2_ single-crystal substrates and epitaxially and laterally overgrown onto Pt NPs, which act as “stepping stones” for electrical conduction by insulator-to-metal transition. **b** Reciprocal space mapping around the (112) reflection of (001) TiO_2_ substrate. Coherently tensile-strained VO_2_ epitaxial films were preserved on both TiO_2_ substrates (*t*_*Pt*_ = 0 s) and Pt NP-TiO_2_ substrates (*t*_*Pt*_ = 5 s). **c** Cross-sectional HAADF-STEM image and elemental EDS mapping of VO_2_ epitaxial films on Pt NP-TiO_2_ substrates (*t*_*Pt*_ = 5 s). **d** Magnified HAADF-STEM image of high-quality epitaxial VO_2_ films on Pt NP-TiO_2_ substrates along the [010] zone axis. **e** Temperature-dependent sheet resistance of VO_2_ epitaxial films without Pt NP incorporation (*t*_*Pt*_ = 0 s, black dotted line) and with Pt NP incorporation (*t*_*Pt*_ = 1–5 s, a series of red solid lines). All VO_2_ films consistently show a steep transition regardless of Pt NP coverage. All Pt-NP-embedded VO_2_ films consistently show a single-crystal-like steep insulator-to-metal transition at identical temperature with hysteresis, which electrically confirms undamaged crystal-quality of fully epitaxial VO_2_ films with randomly oriented metallic Pt NPs.

This electrically triggered IMT induces anisotropic growth of metallic puddles in the domain evolution and a subsequent increase of percolated metallic domains along the direction of the electric field between two electrodes^2,14,16,17,25,26^. Geometric evolution of the switchable (metallic or insulating) domains significantly influences the macroscopic physical properties of VO_2_ (e.g., degree of resistivity modulation, steepness of phase transition); the performance of VO_2_-based threshold switches could be tuned by effectively bridging two electrodes by evolving metallic domains in the insulating matrix (left in Fig. 1a)^2,14,16,17,25,26^. Permanently embedded metal in the switchable VO_2_ matrix^28,29^ may serve as ‘stepping-stones’ to assist the bridging and stabilization of metallic domains during electrically triggered IMT switching dynamics (right in Fig. 1a). However, it is challenging to incorporate metallic nanoparticles (NPs) in single-crystalline VO_2_ without deteriorating the characteristics of IMT due to crystallographic mismatch between the metal and VO_2_.

Here, we demonstrate that embedded metallic NPs promote electric-field-induced metastability of switchable metallic domains in epitaxial VO_2_ thin films (Fig. 1a). After the decoration of uniformly dispersed Pt NPs with different coverage on TiO_2_ substrates, the growth of VO_2_ films allows the selective nucleation on TiO_2_ substrates and subsequent overgrowth on Pt NPs; all Pt-NP-embedded VO_2_ epitaxial films uniquely exhibit a single-crystal-like steep insulator-to-metal transition near room-temperature, which benefits from undamaged crystal quality of fully epitaxial VO_2_ films with metallic Pt NPs. Using this excellent model system to study the influence of a permanent metallic regime on the switchable (metallic or insulating) domains in the VO_2_ matrix, embedded Pt NPs make a significant contribution to reducing the power consumption by decreasing *V*_*th*_ in two-terminal threshold devices. More importantly, embedded Pt NPs are likely to provide 33.3 times longer ‘memory’ of previous super-threshold firing by serving as pre-formed ‘stepping-stones’ between triggered metal domains in the VO_2_ matrix; memory of previous threshold firing is retained for longer than a six orders of magnitude timescale (*τ*_50%_ = 437.88 ms) after the insulating resistance has recovered (*t*_*off*_ = 190 ns) in Pt-NP-embedded VO_2_ epitaxial films. These results emphasize the importance of geometric evolution of the switchable (metallic or insulating) domains in the metal-insulator transition to control macroscopic physical properties by switching dynamics. Moreover, this strategy can be exploited in potential applications of versatile devices for energy-efficient switches (e.g., solid-state frequency discriminator)^2,5^ and for non-Boolean computing (e.g., artificial spiking neurons and synapses using time-dependent plasticity)^4–6^.

## Results & discussion

Without Pt nanoparticle (NP) incorporation (i.e., *t*_*Pt*_ = 0 s), 10 nm-thick (001)_R_-oriented VO_2_ epitaxial films (in rutile notation) were directly grown on (001) TiO_2_ substrates at 400 °C (left in Fig. 1a) by pulsed laser deposition (PLD). X-ray diffraction (XRD) results at 25 °C showed a sharp (002)_R_ rutile VO_2_ peak at ~ 2*θ* = 65.9° without other peaks related to vanadium oxides that had valence states other than +4 (See Supplementary Fig. 1). Moreover, reciprocal space mapping (RSM) around the (112) reflection of the (001) TiO_2_ substrate clearly showed identical in-plane reciprocal space unit of TiO_2_ substrates and VO_2_ films (left in Fig. 1b)^22,30–33^; these results represent the formation of coherently tensile-strained VO_2_ films on TiO_2_ substrates along the in-plane direction with high crystal quality (See Supplementary Fig. 2)^22,30–33^.

To embed metallic NPs into epitaxial VO_2_ films without damaging the crystal quality of VO_2_ films, different density of Pt NPs was provided using sputtering on TiO_2_ substrates by controlling the Pt deposition time (*t*_*Pt*_ ≤ 5 s)^9,31,34^. Since Pt deposition ceased before island coalescence, a cross-sectional high-angle annular dark field (HAADF) image confirms that several nanometers of Pt islands are uniformly dispersed on the TiO_2_ substrates and are disconnected with the adjacent Pt islands (Fig. 1c)^9,31,34^. Then, 10-nm-thick VO_2_ thin films were grown at 400 °C by PLD on Pt NP-decorated (001)_R_-TiO_2_ substrates (denoted as Pt NP-TiO_2_ hereafter) with different coverage of Pt NPs (*t*_*Pt*_ ≤ 5 s) (right in Fig. 1a).

Interestingly, high crystal quality of VO_2_ epitaxial films was preserved on Pt NP-TiO_2_ substrates regardless of the Pt NP decoration with different coverage. A series of RSM data consistently showed identical in-plane reciprocal space unit of strong (112) VO_2_ reflection with TiO_2_ substrates in all VO_2_ films on Pt NP-TiO_2_ (*t*_*Pt*_ = 0, 1, 3, 5 s) (Fig. 1b, See Supplementary Fig. 3); these results implicate the stabilization of coherently strained epitaxial VO_2_ films even on Pt NP-TiO_2_ substrates (right in Fig. 1b)^22,30–33^. The formation of high-quality epitaxial VO_2_ films on Pt NP-TiO_2_ could be confirmed by cross-sectional scanning transmission electron microscopy (STEM) analysis with energy dispersive spectroscopy (EDS) mapping of VO_2_/Pt-NP-TiO_2_ (*t*_*Pt*_ = 5 s) samples (Fig. 1c, d). The bright contrast in HAADF-STEM and the yellow regions in the element-resolved EDS mapping confirm that randomly oriented Pt NPs are uniformly dispersed on the TiO_2_ substrates (Fig. 1c). From magnified HAADF-STEM image at the atomic scale along the [010] zone axis (Fig. 1d), the VO_2_ thin film is epitaxially grown even on the randomly oriented Pt NPs, as well as on TiO_2_ substrates between separated Pt NPs; the in-plane lattice parameter of VO_2_ epitaxial film perfectly matched that of TiO_2_ single-crystal substrates with coherent interfaces, which is consistent with the RSM (Fig. 1b), selective-area diffraction pattern (See Supplementary Fig. 4) and geometric phase analysis for strain (See Supplementary Fig. 5).

To confirm the intact quality of Pt-NP-embedded VO_2_ epitaxial films, temperature-dependent sheet resistance was measured in order to characterize the metal-insulator transition characteristics for VO_2_ epitaxial films with different Pt coverage (0 ≤ *t*_*Pt*_ ≤ 5 s) (Fig. 1e). Due to the increased volume fraction of permanent metallic Pt NPs embedded into the insulating VO_2_ phase, the sheet resistance of the insulating phase (*T* < *T*_*MI*_) decreased with increasing Pt NP coverage from *t*_*Pt*_ = 0 to *t*_*Pt*_ = 5 s. However, regardless of the Pt NP coverage, it should be emphasized that all Pt-NP-embedded VO_2_ films consistently show a steep insulator-to-metal (and metal-to-insulator) transition at identical temperature (*T*_*IM*_ ~ 20 °C, *T*_*MI*_ ~ 14 °C) with hysteresis; this steep transition would be only observed in single-crystal-like VO_2_ films^2,18,30,31^, which confirms on undamaged crystal quality of fully epitaxial VO_2_ films with Pt metallic NPs.

The epitaxy of VO_2_ films on randomly oriented Pt NPs is remarkable because the absence of lattice matching fundamentally limits epitaxial growth of functional layers on underlying layers without crystallographic coordination. Our observation of (001)_R_-VO_2_ epitaxial films both on (001) TiO_2_ substrates and on Pt NPs indicates that VO_2_ crystals initially prefer to nucleate on the TiO_2_ single-crystal substrates, rather than randomly oriented Pt NPs, and then lateral VO_2_ growth was seeded by epitaxial deposits initially formed on exposed regions of the TiO_2_ substrates^35^. Thus, this sequential VO_2_ growth (i.e., selective nucleation on single-crystal substrates + epitaxial lateral overgrowth onto metallic NPs, right in Fig. 1a, See Supplementary Fig. 6) enables transfer of crystal information from TiO_2_ substrates even onto metallic NPs. Due to the selective nucleation and subsequent overgrowth of VO_2_ films, the roughened surface by Pt NPs before the VO_2_ growth (*r*_*RMS*_ = 0.225 nm) was flattened after the VO_2_ growth (*r*_*RMS*_ = 0.124 nm) (See Supplementary Fig. 7).

For heterogeneous nucleation on the substrate during the film growth, the heterogeneous nucleation rate (*N*_*het*_) strongly depends on the activation barrier (ΔG^*^) for the formation of crystalline nuclei (i.e., ${N}_{{het}}\propto {\exp }(-\frac{{\triangle G}^{*}}{{kT}})$). Depending on films and underlying substrates, the activation barriers are determined based on the following expression^36^.

1 $${\triangle G}^{*}=\frac{16\pi {\gamma }^{3}}{3{\Delta G}_{v}}S(\theta )$$

where *ΔG*_*v*_, *γ*, and *S(θ)* are the chemical free energy change for the formation of solid VO_2_ nuclei, interfacial free energies, and a geometrical factor for heterogeneous nucleation, respectively. By comparing the nucleation of VO_2_ films on single-crystal TiO_2_ with that on randomly oriented Pt, ΔG^*^ (VO_2_ on TiO_2_) would be substantially lower than ΔG^*^ (VO_2_ on Pt) due to lower *γ* by the coherent interface between VO_2_ and TiO_2_. Moreover, the sticking coefficient of VO_2_ on single-crystal TiO_2_ is much larger than that on Pt (i.e. VO_2_ nuclei is more easily formed on the TiO_2_ surface than on the Pt surface)^37,38^. Preferential nucleation of VO_2_ films is guided by TiO_2_ single-crystal substrates at the initial growth stage, and then permits subsequent epitaxial lateral overgrowth and coalescence of epitaxial VO_2_ films onto Pt NPs by faster crystal growth oriented in the <110> and <100> direction (i.e. lateral direction of the VO_2_ film) due to a lower surface energy than that of the <001> direction^35,39^.

**Fig. 2:**
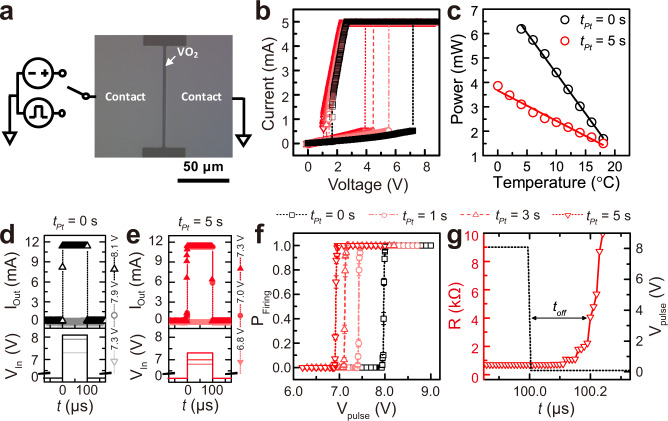
Current–voltage characteristics of two-terminal VO_2_ Mott switches as a function of Pt NP coverage. **a** Schematic representation of the voltage-triggered insulator-to-metal transition (IMT) measurement setup and optical microscope image of VO_2_ Mott switch with 5 $\mu$m electrode separation and 100 $\mu$m electrode width. The performance of VO_2_-based threshold switches was measured with either a continuous voltage sweep or an ultrafast voltage pulse. **b** Continuous voltage sweep of all two-terminal devices with Pt-NP-embedded epitaxial VO_2_ films. **c** The power to turn on a threshold device with *t*_*Pt*_= 0 s and 5 s. Solid lines are the linear best fits to the data, which were extracted from Supplementary Fig. 6. **d** The transient current response to three pulses with different amplitudes (7.3 V, 7.9 V, and 8.1 V) in VO_2_ threshold devices without Pt NP coverage (*t*_*Pt*_ = 0 s). **e** The transient current response to three pulses with different amplitudes (6.8 V, 7.0 V, and 7.3 V) in VO_2_ threshold devices with Pt NP coverage (*t*_*Pt*_ = 5 s). The duration of the pulse is set to 100 $\mu$s. **f** The probability of firing the IMT (*P*_*Firing*_) as a function of the pulse amplitude (*V*_*pulse*_) for each *t*_*Pt*_. *V*_*th,pulse*_ decreased with increasing *t*_*Pt*_. The error bars were calculated using the standard deviation of the binomial distribution. **g** Transient electrical resistance vs. time behavior of VO_2_ threshold devices with Pt NP coverage (*t*_*Pt*_ = 5 s) immediately after the 100 $\mu$s voltage pulse is off at *T* = 12 °C.

Our single-crystal-like VO_2_ films with embedded Pt NPs provide an excellent model system to study the influence of permanent metallic domains (i.e., Pt) on the percolation of switchable (metallic or insulating) domains (VO_2_); this unconventional geometric evolution would strongly influence the performance of VO_2_-based threshold switches utilizing an electric-field-induced steep transition. For this purpose, two-terminal devices were fabricated with an electrode separation of 5 $\mu$m and an electrode width of 100 $\mu$m on Pt-NP-embedded epitaxial VO_2_ films, as shown in optical microscope images (Fig. 2a); narrow electrode separation (~5 $\mu$m) allows the application of a sufficient electric field (~1.7 MV/m) to trigger electrically induced threshold ΙΜΤ switching using several volts^23,40^. As the applied voltage was increased, a sudden increase in current was observed in all two-terminal devices with Pt-NP-embedded epitaxial VO_2_ films (Fig. 2b). This switching is volatile: as the voltage is reduced, the current decreases sharply; all two-terminal devices showed abrupt voltage-triggered threshold ΙΜΤ switching. The threshold voltage (*V*_*th*_) with normal distribution is strongly modulated by coverage of Pt NPs embedded in VO_2_ films (*V*_*th*_ = 6.54 V for *t*_*Pt*_ = 0 s → ${V}_{{th}}$ = 3.80 V for *t*_*Pt*_ = 5 s). (Fig. 2b, See Supplementary Fig. 8).

The reduced *V*_*th*_ for Pt-NP-embedded VO_2_ films is advantageous for reducing the switching power of voltage-triggered IMT. The power to turn on a threshold device (*P*_*ON*_) was calculated according to the following relationship^23^.

2 $${P}_{{ON}}={G}_{{therm}}\left(\Delta T\right)={\left({V}_{{th}}\right)}^{2}/{R}_{{OFF}}$$

where *G*_*therm*_, *R*_*OFF*_, and *V*_*th*_ are the thermal conductance, the electrical resistance below *T*_*IMT*_, and the voltage at which the device turns on, respectively. To investigate the influence of embedded Pt NPs on the VO_2_ threshold switching power, two-terminal IV characteristics were measured as a function of temperature below *T*_*IMT*_ (See Supplementary Fig. 9); *V*_*th*_ also linearly decreased with temperature. The linear *ΔT* dependence of *P*_*ON*_ is characteristic of Joule-heating induced IMT switching. Owing to the more reduced *V*_*th*_ with greater Pt NP coverage, devices with *t*_*Pt*_ = 5 s (e.g., *P*_*ON*_ = 3.103 mW at 4 °C) show much lower power consumption that those with *t*_*Pt*_ = 0 s (e.g., *P*_*ON*_ = 6.195 mW at 4 °C) (Fig. 2c).

Therefore, permanent Pt NPs embedded in the VO_2_ matrix make a significant contribution to reducing the power consumption to induce an electrical conduction pathway between two electrodes in threshold devices. In particular, electric-field-induced Joule heating between two electrodes leads to an abrupt redistribution of local temperature^3,14,26^, which in turn leads to the localized connection of metallic domains along the direction of the electric field (right in Fig. 1a). From a microscopic viewpoint, the decrease of *V*_*th*_ indicates that permanent Pt NPs lower the energy consumption to connect percolated metallic domains by electric-field-induced nucleation and anisotropic growth of switchable metallic domains in VO_2_^3,5,14,25,26^. If permanent metallic Pt NPs are randomly distributed in the VO_2_ matrix, the threshold electric field to nucleate metallic domains in VO_2_ and bridge the electrodes is reduced by shortening the connecting current path: embedded Pt NPs act as stepping-stones for current flow between the electrodes. Moreover, the presence of permanent metallic inclusions creates an inhomogeneous field distribution in the VO_2_ matrix^41^; The electric field in the insulating VO_2_ matrix between Pt metallic NPs is greatly enhanced due to a field-focusing effect; this enhancement locally triggers the IMT at reduced electric field and power consumption.

Facilitated threshold switching by embedded Pt NPs in the epitaxial VO_2_ matrix significantly affects the switching dynamics of the phase transition subjected to ultrafast voltage pulse (Fig. 2d, e, See Supplementary Fig. 10). In particular, exciting the system with a voltage pulse, not a continuous voltage sweep, and monitoring the recovery process provides a probing technique, which enables to capture the dynamic evolution of the switchable domain as a function of time^2,3,5,19,42^. The amplitude of input voltage pulses was modulated from 6.0 V to 9.0 V with 100 *μ*s pulse duration at 12 °C to switch the insulating states to metallic states in a VO_2_ Mott switch with different Pt coverage (*t*_*Pt*_ = 0–5 s) (Fig. 2d, e, See Supplementary Fig. 10).

The characteristics of pulse-triggered threshold switching were clearly demonstrated by an abrupt current response as a function of the input voltage-pulse amplitude near the threshold amplitude (*V*_*th,pulse*_). The gray and black plots in Fig. 2d show the transient current response to three pulses of different amplitude (7.3 V, 7.9 V, and 8.1 V) in VO_2_ threshold devices without Pt NP coverage (*t*_*Pt*_ = 0 s). The distinct difference between them represents the steep threshold characteristics of an electrically triggered IMT by 100 μs pulse: electrical pulse stimuli are insufficient to induce IMT threshold switching if *V*_*pulse*_ < *V*_*th,pulse*_ (*V*_*pulse*_ ~ 7.9 V for VO_2_ (*t*_*Pt*_ = 0 s)), while *V*_*pulse*_ > *V*_*th,pulse*_ (i.e., *V*_*pulse*_ ~ 8.1 V for VO_2_ (*t*_*Pt*_ = 0 s) in Fig. 2d) yields an abrupt increase of current (*I*_*ON*_ / *I*_*OFF*_ > 10^3^), which was limited by an external compliance current. We note that *V*_*th,pulse*_ and *I*_*OFF*_ (i.e., related to the resistance of insulating phase) remained unchanged after more than 100 repetitive firing events, ruling out that device degradation or defect creation is responsible for the effect^16^.

*V*_*th,pulse*_ for a voltage-pulse-triggered IMT was modulated by Pt NP-embedded VO_2_ threshold devices. Despite the universal feature on current amplification at *V*_*pulse*_ > *V*_*th,pulse*_ in all Pt NP-embedded VO_2_ devices, *V*_*th,pulse*_ for a voltage-pulse-triggered IMT was systematically decreased with increasing Pt NP coverage down to 15% (i.e., *V*_*th,pulse*_ = 8.0 V, 7.5 V, 7.1 V, 6.8 V for *t*_*Pt*_ = 0 s, 1 s, 3 s, 5 s, respectively) (see Fig. 2d, e, See Supplementary Fig. 10). These distinct characteristics in *V*_*th,pulse*_ are statistically quantified in Fig. 2f, where the probability of firing the IMT (*P*_*IMT*_) as a function of the pulse amplitude (*V*_*pulse*_) shows a step-like behavior around *V*_*th,pulse*_: *P*_*IMT*_ = 0, where *V*_*pulse*_ < *V*_*th,pulse*_ and *P*_*IMT*_ = 1, where *V*_*pulse*_ > *V*_*th,pulse*_. *V*_*th,pulse*_ for an abrupt threshold conduction decreases with Pt NP coverage (*t*_*Pt*_ = 0 → 5 s); embedded Pt NPs accelerate percolation and bridging of metallic domains by voltage pulse in an ultrafast time regime.

To evaluate how fast this volatile metallic state returns to the insulating state after the voltage pulse ceases, the resistance was monitored immediately after the pulse application of an 8.0 V amplitude and 100 *μs* duration in the VO_2_ Mott switches as a function of time (Fig. 2g (for *t*_*Pt*_ = 5 s), See Supplementary Fig. 11 (for *t*_*Pt*_ = 0 s))^2^. The threshold devices show a transient increase of resistance. Regardless of the existence of Pt NP, resistance remains close to metallic states for ~190 ns (*t*_*off*_; black arrows both in Fig. 2g and in See Supplementary Fig. 11) before rising abruptly. Since *t*_*off*_ can be regarded as the characteristic time for the reverse MIT switching, this *t*_*off*_ provides timescale to lose percolation of the Joule-heating-induced metallic pathway after the removal of the external voltage pulse^2,13,14^.

**Fig. 3:**
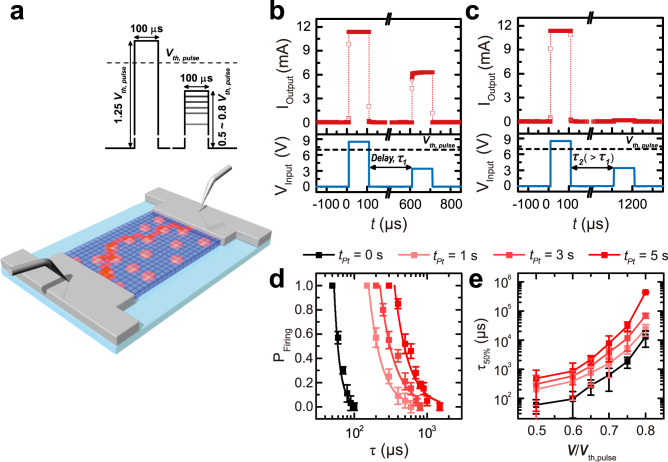
Enhancement of sub-threshold firing and memory effect in Pt-NP-embedded VO_2_. **a** Conceptual illustration of percolative (sub-)threshold firing of Pt-NP-embedded VO_2_ Mott switches by consecutive electrical pulses. Even if the second probe pulse is applied below *V*_*th,pulse*_, the metallic domain (red) can be connected by IMT switching from insulating domains (blue) assisted by the permanent metallic Pt ‘stepping stone’ (coral) due to the percolative nature of the IMT, as long as a certain delay time (*τ*) for the relaxation is short enough to ‘memorize’ the previous firing event by the super-threshold pump pulse. **b**, **c** Transient current output by two consecutive voltage pulses with different delay time measured by a pump-probe procedure at *T* = 12 °C: a super-threshold pulse is firstly applied to trigger the insulator-to-metal transition, followed by a sub-threshold probe pulse, after a delay time *τ*. (**b** *τ*_*1*_ = 500 $\mu$s and **c** *τ*_*2*_ = 1000 $\mu$s between the two consecutive pulses). **d** Probability that the probe pulse will trigger the insulator-to-metal transition (*P*_*Firing*_) as a function of *τ* at *T* = 12 °C depending on Pt NP coverage. *P*_*Firing*_ is plotted for *V*_*probe*_ = 0.5 *V*_*th,pulse*_ with different Pt NP coverages: *t*_*Pt*_ = 0 s, 1 s, 3 s, and 5 s. This probability was obtained after averaging 100 pump–probe measurements at each *τ*. **e** Delay time at which the sub-threshold firing probability is 50% (τ_50%_) plotted against *V*_*probe*_/*V*_*th,pulse*_ at 12 °C. τ_50%_ was calculated using the fitting curves shown in **d**. The error bars in **d** and **e** were calculated using the standard deviation of the binomial distribution.

Due to the percolative nature of IMT and MIT during the application and removal of the input voltage pulse, respectively, the current output shows a distinct response by consecutive pump-probe pulses (Fig. 3a)^2,5^. In particular, even if the second probe pulse is applied below *V*_*th,pulse*_, metallic output current can be triggered (i.e., sub-threshold firing), as long as certain delay time ($\tau$) for the relaxation is short enough to ‘memorize’ the previous firing event by the preceding super-threshold pump pulse (Fig. 3). Since ‘sub-threshold firing’ is governed by the characteristic time of phase relaxation, a pump-probe procedure by electric pulses was utilized to investigate how this percolative systems relaxes. For example, a ‘super-threshold’ pump pulse with amplitude *V*_*pump*_ = 1.25 *V*_*th,pulse*_ and duration of 100 μs was firstly applied to excite the VO_2_ films, and metallization was thereby triggered. Then, after a different delay time (*τ* = 500 μs and 1000 μs) for the relaxation, a second voltage pulse (probe) was sent with sub-threshold amplitude (*V*_*probe*_ < *V*_*th,pulse*_) and 100 μs duration (Fig. 3b, c).

It is possible to re-trigger the IMT by using low sub-threshold voltage pulse (*V*_*probe*_ = 0.5 *V*_*th,pulse*_) in a much longer time interval between pulses (*τ*_1_ = 500 μs) in Pt-NP-embedded VO_2_ devices (Fig. 3b). With a single pulse, this subthreshold pulse should not trigger the IMT; it should be emphasized that this *V*_*probe*_ is much lower than the *V*_*th,pulse*_, and is in contrast to the non-firing behavior under single pulse applications. This ‘memory’ of the previous firing was lost at a pump-probe time interval of *τ*_2_ = 1000 μs (> *τ*_1_) (Fig. 3c). This result indicates that the device maintains a certain ‘memory’ of the previous firing event and thus facilitates the firing again with sub-threshold pulses^2,5^.

Interestingly, the delay time (*τ*) between pump and probe pulses is 500 $\mu$s, which is more than three orders of magnitude larger than the electrically measured MIT recovery time (*t*_*off*_ *~* 190 ns). Metal-to-insulator recovery after removal of the voltage pulse corresponds to the rupture or depercolation process of already formed metallic filaments^2,5,13,14,24^. The evolution of subthreshold firing at *τ ≫ t*_*off*_ indicates that memory of previous super-threshold switching is retained long after the insulating resistance has recovered. From a microscopic viewpoint, metallic domain puddles are likely to exist for a much longer time in the localized area after the rupture and depercolation process of the pre-formed metallic filament for electric conduction by the preceding threshold *V*_*pump*_.

To investigate the influence of Pt NP coverage on this characteristic time (*τ*) of ‘memory’ of the metallic domain, we carried out a pump-probe experiment of VO_2_ threshold devices with different Pt NP coverage (*t*_*Pt*_ = 0–5 s) by changing the pulse separation time (*τ*) (Fig. 3d, e). The probability for the second probe pulse to trigger the metallic conduction is shown in Supplementary Fig. 12 as a function of *τ* for different amplitudes of *V*_*probe*_. Since ‘memory’ of metallic triggering is likely to be lost as the pulse separation time (*τ*) increases, the probability consistently decreases with *τ* regardless of the Pt coverage (Fig. 3d). By defining *τ*_50%_ as the delay time for which the firing probability declines to 50%, *τ*_50%_ increases with amplitude of *V*_*probe*_ (Fig. 3e) and temperature (See Supplementary Fig. 13); the increase of the second pulse amplitude substantially enhances the success probability for subthreshold firing (Fig. 3e). More importantly, as Pt NP coverage increases (*t*_*Pt*_ = 0 s → *t*_*Pt*_ = 5 s), *τ*_50%_ increases by up to 33.3 times longer at the same *V*_*probe*_ (e.g., *τ*_50%_ = 13.15 ms (for *t*_*Pt*_ = 0 s) → *τ*_50%_ = 437.88 ms (for *t*_*Pt*_ = 5 s) at *V*_*probe*_ = 0.8 *V*_*th,pulse*_) (Fig. 3e). It should be emphasized that the memory of previous threshold firing is retained for longer than a six orders of magnitude timescale (*τ*_50%_ = 437.88 ms) after the insulating resistance has recovered (*t*_*off*_ = 190 ns) in Pt-NP-embedded VO_2_ epitaxial films.

Therefore, embedded Pt NPs in the VO_2_ matrix are likely to enhance the ‘memory’ of previous firing as pre-formed ‘stepping-stones’ between fired metal domains in VO_2_; these permanent metallic ‘stepping-stones’ significantly facilitate sub-threshold firing in the consecutive pulses. Moreover, metallic domain puddles are ruptured (or disconnected) after the removal of the preceding ‘super-threshold’ pulse; these metastable metallic puddles are likely to remain trapped longer in Pt NP-embedded VO_2_ films than in pure VO_2_ films. Thus, these persistent and long-lived domains after the preceding threshold switching act as nuclei that facilitate triggering of the subsequent IMT with a subthreshold voltage pulse; persistent metallic domains enhanced by permanent Pt NPs are indeed responsible for the longest memory effect in the Pt NP-embedded VO_2_ films (*t*_*Pt*_ = 5 s).

The microscopic origin of persistent metallic domains in the Pt NP-embedded VO_2_ films is attributed into electron doping by the charge transfer from Pt. The lowered work function of Pt NPs (~4.6 eV)^29^ than VO_2_ (~5.0 eV) leads to charge carrier injection from small Pt NPs into VO_2_ matrix by Fermi level alignment between Pt NP and VO_2_, forming an electron rich region in the VO_2_ matrix near the Pt NP contact interface; this ‘local’ electron doping near Pt NPs may result in the local stabilization of ‘persistent metallic domain’; these persistent and long-lived ‘metastable’ metallic domains after the super-threshold switching act as nuclei that lower the activation barrier and facilitate re-triggering of the subsequent IMT with a subthreshold voltage pulse.

**Fig. 4:**
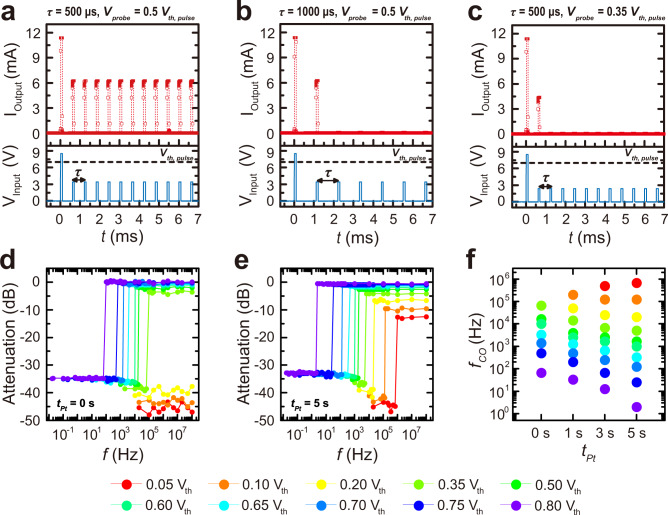
Frequency discrimination using sub-threshold firing of multiple pulses in VO_2_ Mott switches with different Pt NP coverage. **a**–**c** Transient current output (top panels) by multiple consecutive voltage pulses with different pulse separation (or frequency, bottom panels) at *T* = 12 °C: The preceding super-threshold pulse is followed by a series of subthreshold voltage pulses separated by 500 $\mu$s (**a**, **c**) or 1000 $\mu$s. **b** with probe voltage amplitude of *V*_*probe*_ = 0.5*V*_*th,pulse*_ (**a**, **b**) or *V*_*probe*_ = 0.35*V*_*th,pulse*_ **c**. Note that when the pulse separation is 500 $\mu$s at *V*_*probe*_ = 0.5*V*_*th,pulse*_, each voltage pulse refreshes the memory of the device, allowing for repeated subthreshold firing. **d**, **e** Attenuation of a pulsed signal through a device as a function of the signal frequency. Several signal amplitudes are shown with **d**, *t*_*Pt*_ = 0 s and **e**, *t*_*Pt*_ = 5 s. **f** Cut-off frequency (*f*_*CO*_) of Pt-NP-embedded VO_2_ Mott switches plotted against each Pt NP coverage (*t*_*Pt*_) with several signal attenuation levels.

The dependence of sub-threshold firing probability on *τ* could be exploited as a high-pass filter (i.e., frequency discriminator)^2,5^. The super-threshold pulse is followed by a series of sub-threshold pulses separated by *τ*, which determines the frequency (*f*) of electrical stimuli (Fig. 4a–c). Repetitive subthreshold pulses with different *f* were applied after the super-threshold pulse through two-terminal devices with Pt NP-embedded VO_2_ films. Each sub-threshold pulse refreshes the memory of the devices, allowing for continuous subthreshold firing and signal transmission at a high pulse frequency (e.g., *τ* = 500 μs (*f* = 2 kHz), *V*_*probe*_ = 0.5 *V*_*th,pulse*_ for VO_2_ with *t*_*Pt*_ = 5 s in Fig. 4a) at *f* higher than the cut-off frequency (*f*_*CO*_); subthreshold firing ceases at *f* < *f*_*CO*_ (e.g., *τ* = 1000 μs (*f* = 1 kHz), *V*_*probe*_ = 0.5 *V*_*th,pulse*_ for VO_2_ with *t*_*Pt*_ = 5 s in Fig. 4b) or at lower *V*_*probe*_ (e.g., *τ* = 500 μs (*f* = 2 kHz), *V*_*probe*_ = 0.35 *V*_*th,pulse*_ for VO_2_ with *t*_*Pt*_ = 5 s in Fig. 4c). Consequently, clear and sharp (almost 35 dB) high-pass filter characteristics were observed with *V*_*probe*_-dependent *f*_*CO*_ in all devices (Fig. 4d, e); these characteristics show the possibility of tuning the *f*_*CO*_ for signal transmission by adjusting the amplitude of the incoming sub-threshold signal: a lower amplitude of repetitive *V*_*probe*_ leads to a higher *f*_*CO*_.

Notably, Pt NP-embedded VO_2_ devices (*t*_*Pt*_ = 5 s) enable a wider range of *f*_*CO*_ tuning (*f*_*CO*_ = 2 Hz at *V*_*probe*_ = 0.8 *V*_*th,pulse*_ to *f*_*CO*_ = 670 kHz at *V*_*probe*_ = 0.05 *V*_*th,pulse*_) compared to that from pure VO_2_ devices (*t*_*Pt*_ = 0 s) (*f*_*CO*_ = 67 Hz at *V*_*probe*_ = 0.8 *V*_*th,pulse*_ to *f*_*CO*_ = 67 kHz at *V*_*probe*_ = 0.35 *V*_*th,pulse*_) (Fig. 4f). The enhanced *τ*_50%_ of long-lived metallic domains in Pt-embedded VO_2_ films leads to subthreshold firing at a lower frequency (higher *τ*) of repetitive electrical pulse signal; embedded Pt NPs give rise to enhanced connectivity of repetitive stimuli. Moreover, reduced switching power in Pt-embedded VO_2_ films contributes to subthreshold firing at a lower *V*_*probe*_ amplitude (e.g. *V*_*probe*_ = 0.2 *V*_*th,pulse*_ was not subthreshold fired at the Pt-embedded VO_2_ with *t*_*Pt*_ = 0 s but fired at the Pt-embedded VO_2_ with *t*_*Pt*_ = 5 s); embedded permanent metallic NPs bring stable percolation of switchable metallic domains at lower stimuli; a delayed memory effect of switchable metallic domain in VO_2_ films by Pt metallic ‘stepping-stones’ leads to *f*-tunable subthreshold firing in a wider *f* range.

In summary, voltage-triggered metastability of switchable metallic domains was enhanced by embedded metallic NPs that serve as ‘stepping-stones’ in a switchable epitaxial VO_2_ matrix. The ability to selectively nucleate epitaxial VO_2_ films on TiO_2_ substrates, rather than uniformly dispersed Pt NPs, enables a single-crystal-like steep insulator-to-metal transition near room-temperature with different coverage of permanent metallic NPs in the switchable matrix. Using this distinct model system, we systematically investigated the effect of permanent metallic domains on the dynamics of switchable (metallic or insulating) domains in VO_2_ matrix. In particular, embedded Pt NPs make a significant contribution to better inscribed ‘memory’ of previous firing by serving as pre-formed stepping-stones between triggered metal domains in the VO_2_ matrix. In consecutive input pulses, remarkably, memory of previous super-threshold firing is kept for longer than a six orders of magnitude timescale (*τ*_50%_ = 437.88 ms) even after the insulating resistance recovered (*t*_*off*_ = 190 ns) in Pt-NP-embedded VO_2_ epitaxial films.

These characteristics could be used to implement functionalities in oxide electronics. Our results demonstrate that a high-pass filter (i.e., frequency discriminator) can be simply tuned by modified intrinsic dynamics of a percolative phase transition, which is assisted by embedded Pt NP coverage in the VO_2_ matrix. Our results clarify the influence of permanent metallic domains on geometric evolution of switchable (metallic or insulating) domains in a metal-insulator transition. From a practical viewpoint, this strategy to exploit the enhanced ‘memory’ of previous firing by uniformly distributed metallic stepping-stones could open up potential applications of versatile devices for energy-efficient switches^2,5^ and for non-Boolean computing^4–6^.

## Methods

### Epitaxial growth of Pt nanoparticle embedded VO_2_ thin films

Different density of nano-sized Pt islands was formed by controlling the Pt deposition time (0 s ≤ *t*_*Pt*_ ≤ 5 s) on (001) TiO_2_ single crystals at room temperature by sputtering. Since Pt deposition ceased before island coalescence, cross-sectional high-angle annular dark field (HAADF) in scanning transmission electron microscopy (STEM) confirms that several nanometer Pt islands are uniformly dispersed on the TiO_2_ substrates and are disconnected from the adjacent Pt nanoparticles (NP). Then, 10-nm-thick VO_2_ thin films were grown at 400 °C on Pt NP-decorated (001)_R_-TiO_2_ substrates with different coverage of Pt NPs (0 s ≤ *t*_*Pt*_ ≤ 5 s) by pulsed laser deposition with the base pressure of the growth chamber set at ~10^−6 ^Torr. A KrF excimer laser (*λ* = 248 nm) was focused onto a stoichiometric V_2_O_5_ rotating target at fluence ~1.5 J cm^−2^ pulse^−1^ and a repetition rate of 2 Hz. The VO_2_ films were grown in oxygen ambient of 10 mTorr and at a growth temperature of 400 °C to optimize electrical properties with a steep metal-insulator transition. After growth, the sample were cooled to room temperature at 2 °C min^−1^. By optimizing the growth conditions, high crystal quality of VO_2_ epitaxial films was confirmed by a series of reciprocal space mapping data and a steep insulator-to-metal (and metal-to-insulator) transition at identical temperature (*T*_*IM*_ ~20 °C, *T*_*MI*_ ~14 °C) regardless of Pt NP coverage (0 ≤ *t*_*Pt*_ ≤ 5 s) on the TiO_2_ substrate.

### Fabrication of two-terminal threshold Mott devices

Two Pt (50 nm) electrodes with 100 μm lateral width were patterned on top of a Pt-NP-embedded VO_2_ epitaxial film using photolithography and sputtering. A 5-μm gap was left between two electrodes; narrow electrode separation (~5 $\mu$m) allows the application of a sufficient electric field (~ 1.7 MV/m) to trigger electrically induced threshold ΙΜΤ switching using several volts.

### Structural characterization

2theta-omega scan and reciprocal space mapping (RSM) around the (112) TiO_2_ reflection were performed to characterize the crystal quality and lattice parameters in all Pt-NP-embedded VO_2_ epitaxial thin films using a high-resolution X-ray diffractometer (HRXRD, Bruker Discover 8) with Cu K_α1_ radiation (λ = 0.15406 nm) at Materials Imaging & Analysis Center of POSTECH.

For the cross-sectional analysis on VO_2_ epitaxial films on Pt NP-TiO_2_ substrates, the thin foil was prepared by a dual-beam focused ion beam system (Helious Nanolab, Thermo Fisher Co., USA) through the [010] projection. HRTEM, STEM and atomic-scale EDS analyses were performed via the aberration-corrected STEM (JEM-ARM200F, JEOL, Japan) at 200 kV equipped with a fifth-order spherical aberration corrector (ASCOR, CEOS GmbH, Germany) and the dual100 mm^2^ Energy Dispersive X-ray Spectrometer detector (JED-2300T EDS, JEOL, Japan) at the Materials Imaging & Analysis Center of POSTECH. The electron probe for STEM observation was set to be ~70 pm; and the collection semi-angle was ranged from 54 to 216 mrad for High Angle Annular Dark Field (HAADF) imaging. The raw STEM data were filtered to reduce the background scanning noise by using Local 2D Difference Filter (Filters Pro, HREM Research Inc., Japan). EDS mapping signals were obtained during several tens of minutes by the multiple frame summation, up to ~4000 frames of 256 × 256 pixel-resolution; and the acquisition time per a single pixel was set to be 10 μsec. From the atomic scale TEM/STEM images, the strain analysis results were extracted by using the commercial plug-in software (GPA, HREM Research Inc., Japan).

### Electrical measurement

Temperature-dependent sheet resistance was obtained to characterize metal-insulator transition characteristics for VO_2_ epitaxial films with different Pt coverage (0 ≤ *t*_*Pt*_ ≤ 5 s) by the Van der Pauw method. The electrical characteristics of the two-terminal VO_2_ electrical devices were measured using a semiconductor device analyzer (B1500A, Agilent) with a source measurement unit (SMU) and a waveform generator/fast measurement unit (WGFMU) in a temperature-variable probe station under Ar ambient conditions. Current–voltage characteristics were measured by sweeping the voltage from 0 V to 10 V with a 10 mV step using two SMUs on two electrodes. For pulse measurement, WGFMU was utilized to create the input voltage pulses and high-speed probe. Single pulse voltage was generated to monitor the threshold voltage pulse (*V*_*th,pulse*_) by changing pulse the amplitude from 6.0 V to 9.0 V and a pulse duration of 100 *μ*s. To investigate the influence of Pt NP coverage on this characteristic time (*τ*) of ‘memory’ of the metallic domain, a pump-probe experiment of VO_2_ threshold devices was carried out using consecutive input pulses (i.e., preceding super-threshold pump pulse (*V*_*pulse*_ > *V*_*th,pulse*_) and second sub-threshold probe pulse (*V*_*pulse*_ < *V*_*th,pulse*_)) with a pulse duration of 100 *μ*s as a function of different pulse separation time (*τ*). Finally, for a high-pass filter (i.e., frequency discriminator), the super-threshold pulse is followed by a series of repetitive sub-threshold pulses separated by *τ*, which determines the frequency (*f*) of electrical stimuli, with a pulse duration of 100 *μ*s.

## Supplementary information

Supplementary Information

## Data Availability

The authors declare that all the data supporting the finding of this study are available within this article and its Supplementary Information files and are available from the corresponding author on reasonable request.
