# Supplementary material for: Embedded metallic nanoparticles facilitate metastability of switchable metallic domains in Mott threshold switches
Source: Nat Commun. 2022 Aug 10;13:4609. doi: 10.1038/s41467-022-32081-x (PMC9365788; doi:10.1038/s41467-022-32081-x)
Supplement: Supplementary file 1 — Supplementary Information [file 41467_2022_32081_MOESM1_ESM.pdf]

*Supplementary information for*

# Embedded metallic nanoparticles facilitate metastability of switchable metallic domains in Mott threshold switches

Minguk Jo[1)], Ye-Won Seo[1)], Hyojin Yoon[1), 2)], Yeon-Seo Nam[1)], Si-Young Choi[1)], Byung Joon Choi[3)] and Junwoo Son[1),*]

1) Department of Materials Science and Engineering (MSE), Pohang University of Science and Technology (POSTECH), Pohang 37673, Republic of Korea

2) Department of Chemical Engineering and Materials Science (CEMS), University of Minnesota, Minneapolis, MN 55455, United States

3) Department of Materials Science and Engineering (MSE), Seoul National University of Science and Technology (Seoultech), Seoul 01811, Republic of Korea

*jwson@postech.ac.kr

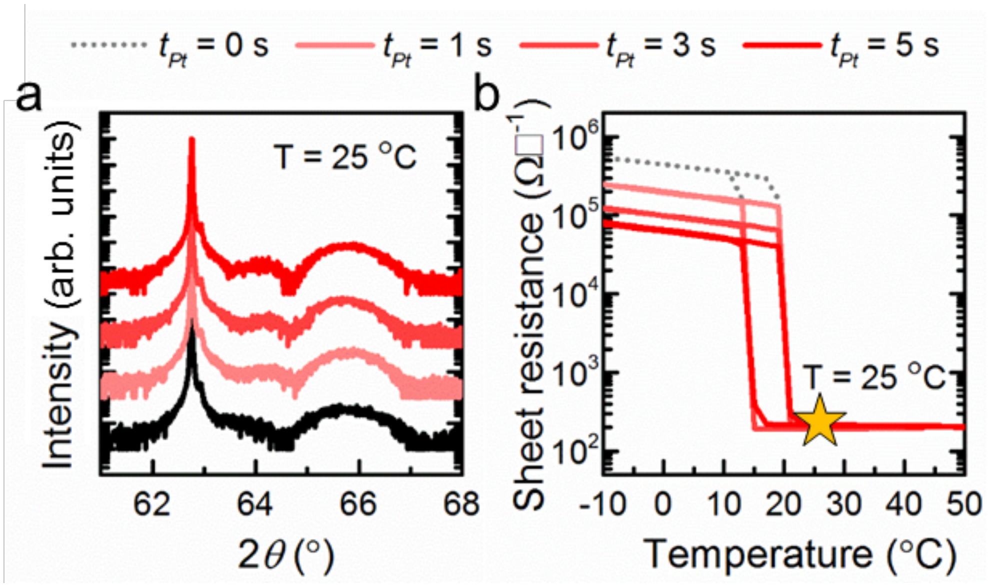


**Supplementary Figure 1 | Symmetrical XRD 2theta-omega scan and temperature-dependent sheet resistance of 10-nm-thick epitaxial $VO_2$ films embedded Pt nanoparticles. a,** 2theta-omega scan of a $VO_2$ film without Pt nanoparticles (NPs) (black line) and with embedded Pt NPs (red series lines) at ~ 25 °C (i.e., room temperature). X-ray diffraction (XRD) results showed a sharp $(002)_R$ $VO_2$ peak at ~ $2\theta = 65.9°$ without other peaks related to vanadium oxides that had valence states other than +4. The appearance of identical $VO_2$ XRD peaks regardless of Pt NP coverage represents that Pt metallic NPs are successfully embedded in epitaxial $VO_2$ films without damaging the crystal quality of $VO_2$ films. **b,** temperature-dependent sheet resistance of 10-nm-thick epitaxial $VO_2$ films embedded Pt nanoparticles. Since all $VO_2$ films show steep insulator-to-metal (and metal-to-insulator) transition at identical temperature ($T_{IM}$ ~ 20 °C, $T_{MI}$ ~ 14 °C) with hysteresis as shown in temperature-dependent sheet resistance (**Fig. 1d**), the sheet resistance is sufficiently low (~ 190 Ohm/sq.) as a metallic rutile phase at 25 °C, which is higher temperature than $T_{IM}$ (~ 20 °C) (see yellow asterisk in **Fig. S1b**)

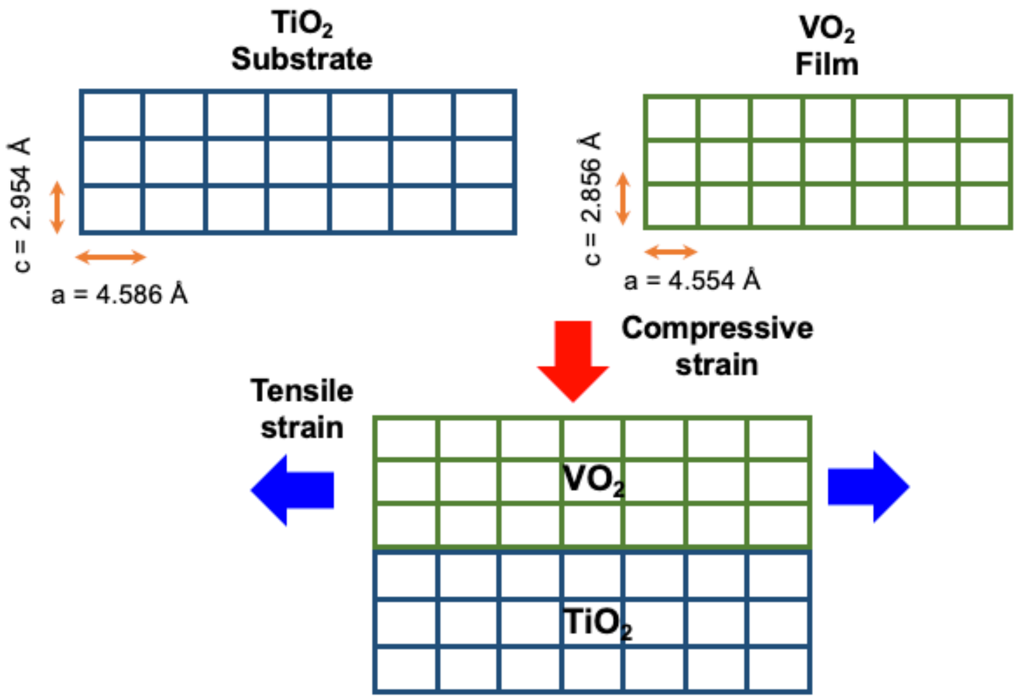


**Supplementary Figure 2 |** The in-plane lattice parameter of $VO_2$ (a = 4.554 Å) is smaller than that of $TiO_2$ (a = 4.586 Å), so the $VO_2$ film on $TiO_2$ substrates would be tensile-strained, rather than compressive-strained, along the in-plane direction (i.e., compressive-strained along out-of-plane direction); the formation of coherently tensile-strained $(001)_R$-oriented $VO_2$ films leads to the lowering of metal-insulator transition temperature (68 °C → 20 °C).

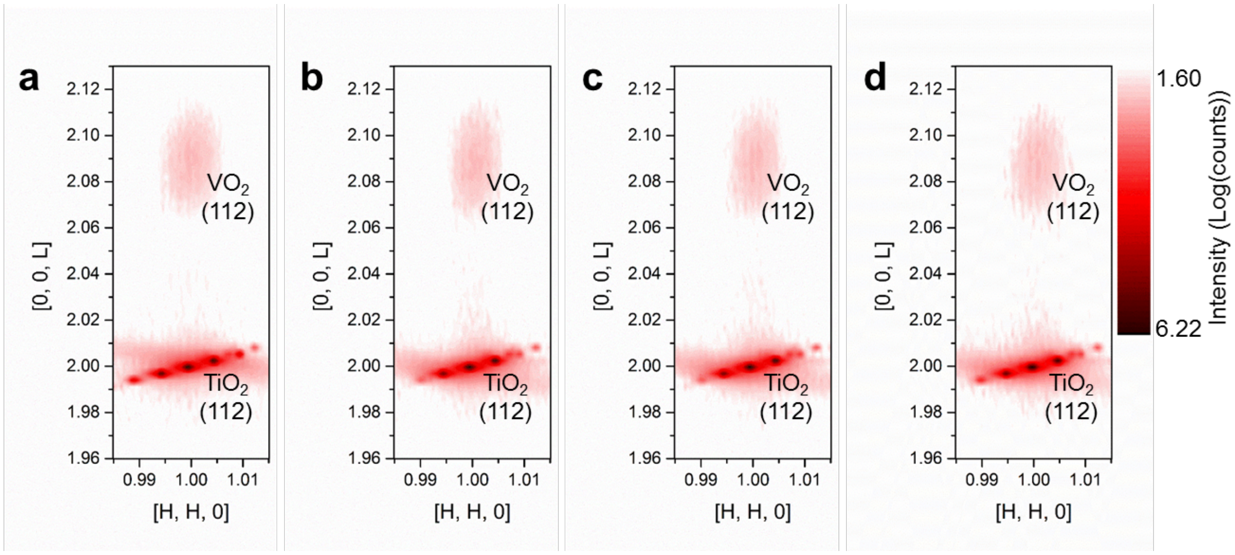


**Supplementary Figure 3 | Reciprocal space mapping (RSM) of 10-nm-thick epitaxial $VO_2$ films with different Pt coverages.** RSM around (112) reflection of (001) $TiO_2$ substrate and the $VO_2$ films without Pt NPs (**a**) and with embedded Pt NPs with different coverages; $t_{Pt}$ = 1 sec (**b**), 3 sec (**c**) and 5 sec (**d**). A series of RSM data consistently showed identical H (i.e., in-plane reciprocal space unit) of strong (112) $VO_2$ reflection with $TiO_2$ substrates in all $VO_2$ films on Pt NP-$TiO_2$ ($t_{Pt}$ = 0, 1, 3, 5 sec); these results implicate the stabilization of coherently strained epitaxial $VO_2$ films even on Pt NP-$TiO_2$ substrates. Thus, high crystal quality of $VO_2$ epitaxial films was preserved on Pt NP-$TiO_2$ substrates regardless of the Pt NP decoration with different coverage.

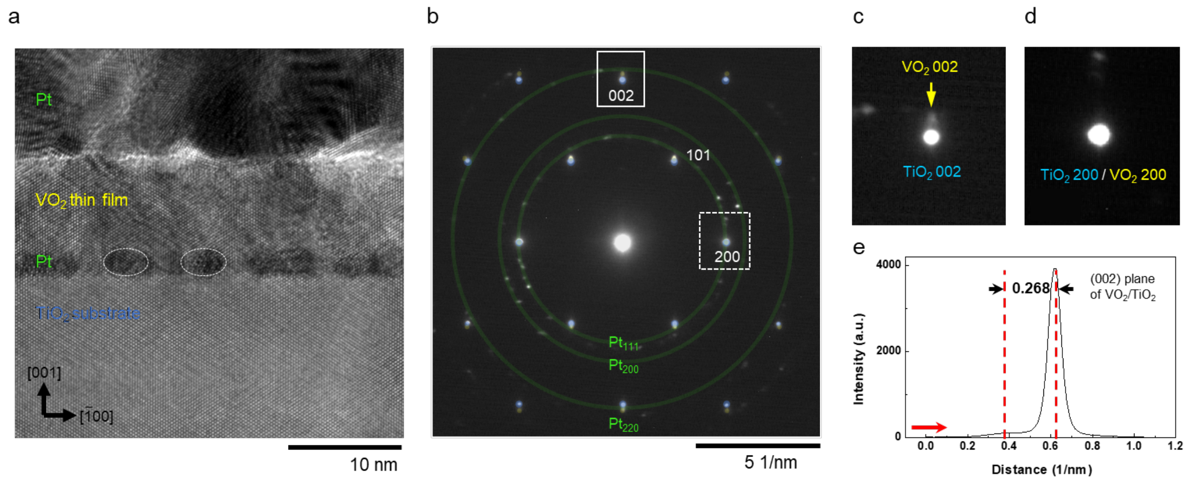


**Supplementary Figure 4 | HRTEM and SADP of Pt-nanoparticle-embedded $VO_2$ epitaxial thin films a,** Cross-sectional HRTEM image at the film and substrate interface. The HRTEM image shows that 10 nm-thick $VO_2$ films were grown on Pt-NP-$TiO_2$ substrates. **b,** SADP image for the $VO_2$ thin film and $TiO_2$ substrate, the diffraction spots from the $VO_2$($TiO_2$) marked by blue(yellow) circles. Ring diffraction patterns from polycrystalline Pt are marked by green lines. **c** and **d,** High magnification images from out-of-plane diffraction patterns(c) and in-plane diffraction patterns(d). The out-of-plane direction diffraction spots are slightly separated, whereas the in-plane direction diffraction spots are completely overlapped as a single spot. It shows that the $VO_2$ thin films are coherently growth on Pt NP-$TiO_2$ substrate **e,** Intensity profile taken from the indicated line in (c). The separation length between $VO_2$ 002 and $TiO_2$ 002 spots is almost same with the calculated; it means $VO_2$ film are compressively strained along the c-axis by biaxial tensile strain along the in-plane direction.

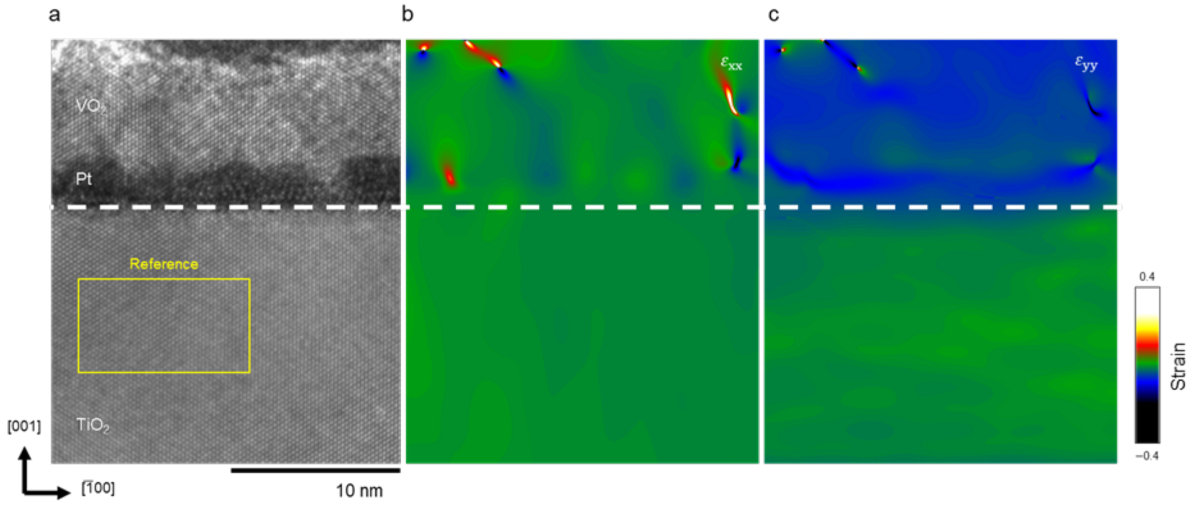


**Supplementary Figure 5 | Strain mapping of the $VO_2$/Pt NP-$TiO_2$ interface. a,** Cross-sectional HRTEM image of $VO_2$/Pt NP-$TiO_2$ interface. **b,** In-plane strain($\epsilon_{xx}$) mapping of (a). **c,** Out-of-plane($\epsilon_{yy}$) strain mapping of (a). These strain maps obtained from geometric phase analysis (GPA) were calculated by the reference region in $TiO_2$ substrate (a yellow box in **a**). It shows that in-plane lattice parameter between the $VO_2$ film and the $TiO_2$ substrate were almost no lattice constant difference, whereas out-of-plane lattice parameters of $VO_2$ are smaller than that of $TiO_2$ substrates.

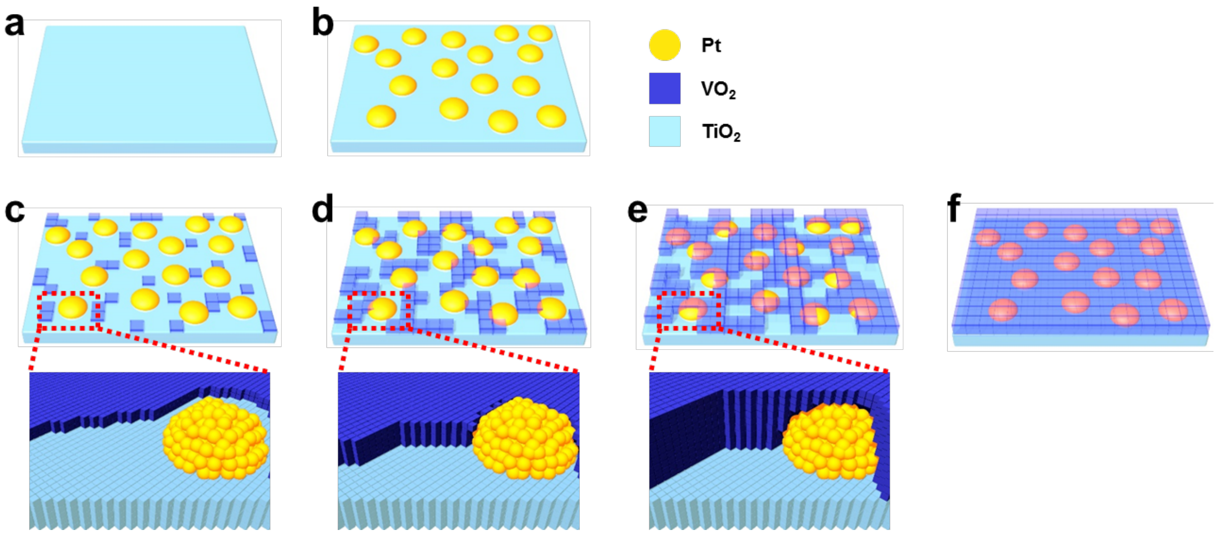


**Supplementary Figure 6 | Schematic representation on sequential growth of epitaxial $VO_2$ films (i.e., selective nucleation on single-crystal substrates + epitaxial lateral overgrowth onto metallic Pt NPs).** Our observation of $(001)_R$-$VO_2$ epitaxial films both on (001) $TiO_2$ substrates and on Pt NPs indicates that $VO_2$ crystals initially prefer to nucleate on the $TiO_2$ single-crystal substrates, rather than amorphous Pt NPs, and then lateral $VO_2$ growth was seeded by epitaxial deposits initially formed on exposed regions of the $TiO_2$ substrates.

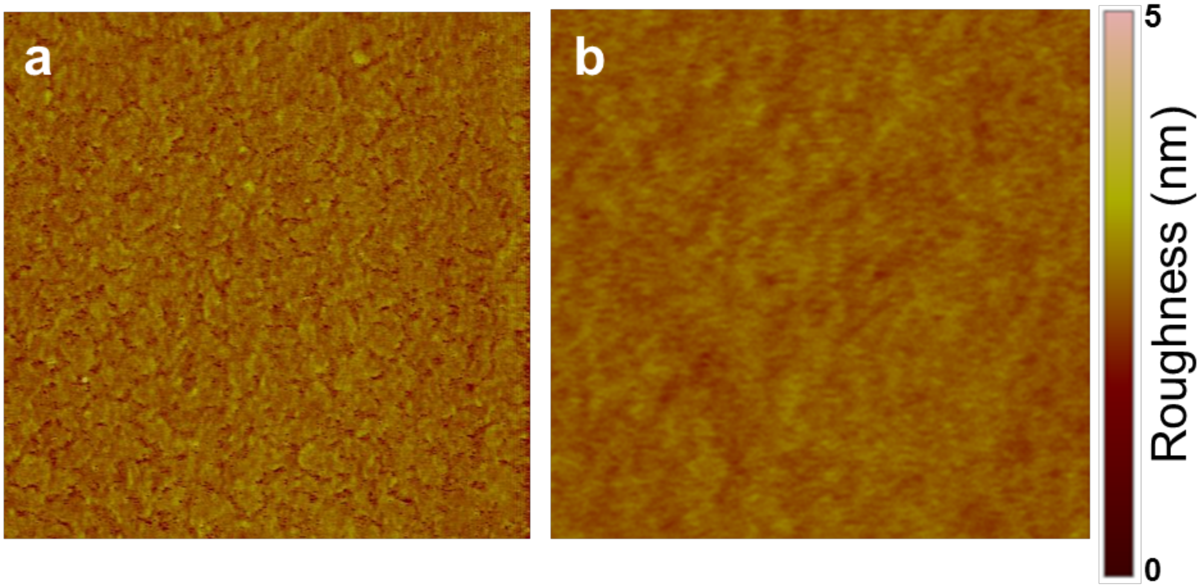


**Supplementary Figure 7 | Atomic forced microscopy a, before and b, after $VO_2$ film growth on Pt-decorated $TiO_2$ substrates.** The roughened surface by Pt NPs ($t_{Pt}$ = 5 sec) before the $VO_2$ growth ($r_{RMS}$ = 0.225 nm) was flattened by epitaxial $VO_2$ films after the $VO_2$ growth ($r_{RMS}$ = 0.124 nm)

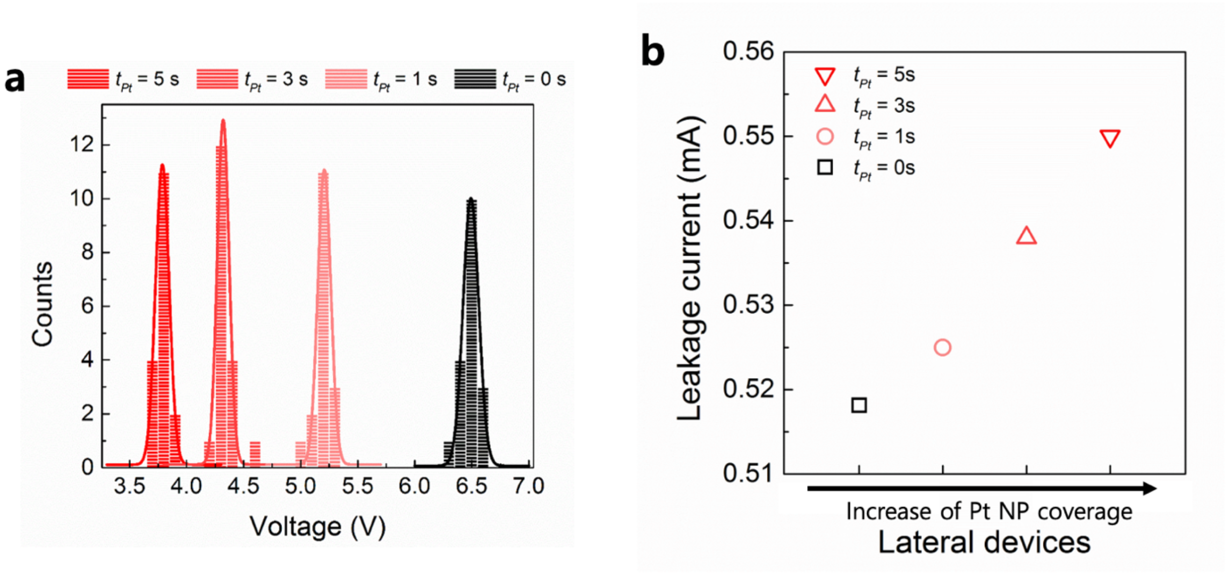


**Supplementary Figure 8 | a,** $V_{th}$ distribution and **b,** leakage current of Pt-NP-embedded $VO_2$ threshold switches as a function of Pt NP coverage.

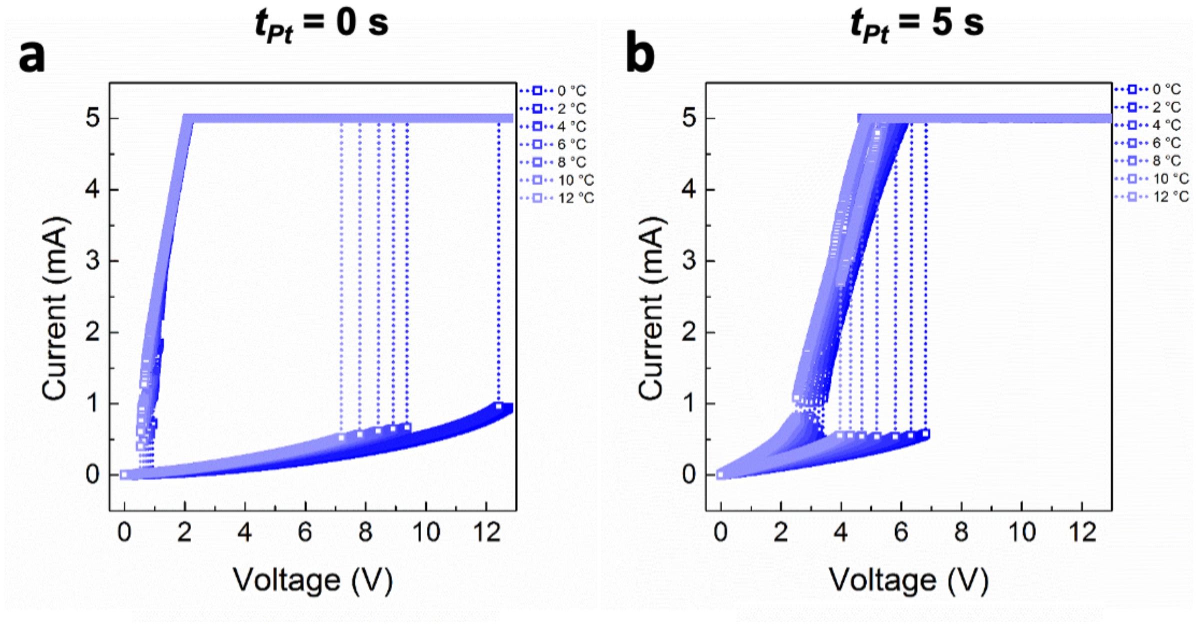


**Supplementary Figure 9 | Temperature-dependent current-voltage characteristics of two-terminal $VO_2$ threshold switches with different Pt NP coverage.** To investigate the influence of embedded Pt NPs on the $VO_2$ threshold switching power (**Fig. 2c**), two-terminal IV characteristics were measured as a function of temperature from 0 °C to 12 °C for devices with $t_{Pt}$ = 0 sec (**a**) and 5 sec (**b**).

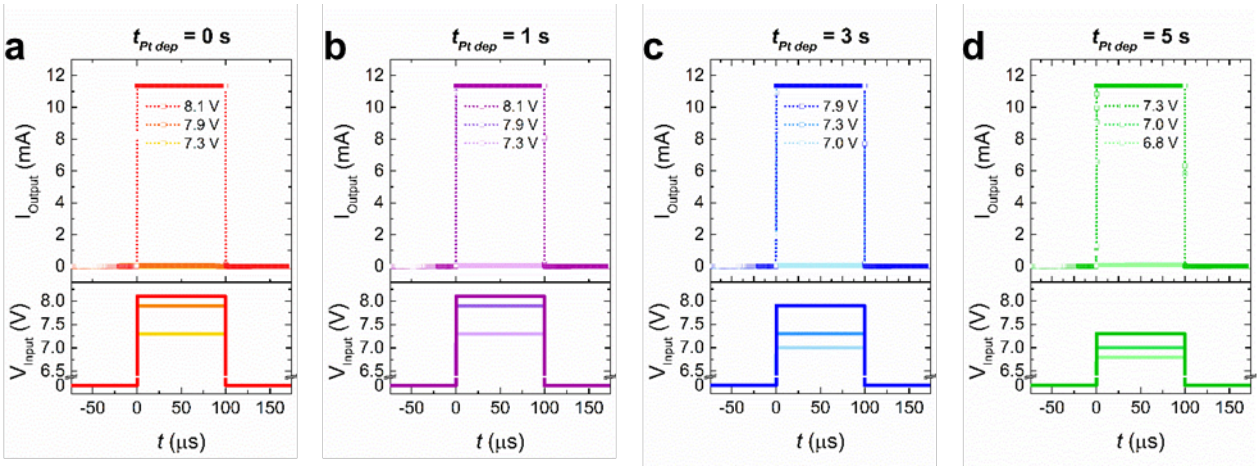


**Supplementary Figure 10 | Voltage-pulse-triggered insulator-to-metal transition (IMT) of $VO_2$ threshold devices with different Pt NP coverages.** The amplitude of input voltage pulses was modulated from 6.0 V to 9.0 V with 100 $\mu$s pulse duration at 12 °C to switch the insulating states to metallic states in a $VO_2$ Mott switch with different Pt coverage (**a,** $t_{Pt}$ = 0 sec, **b,** $t_{Pt}$ = 1 sec, **c,** $t_{Pt}$ = 3 sec, **d,** $t_{Pt}$ = 5 sec). Obviously, $V_{th,pulse}$ for a voltage-pulse-triggered IMT was modulated by Pt NP-embedded $VO_2$ threshold devices.

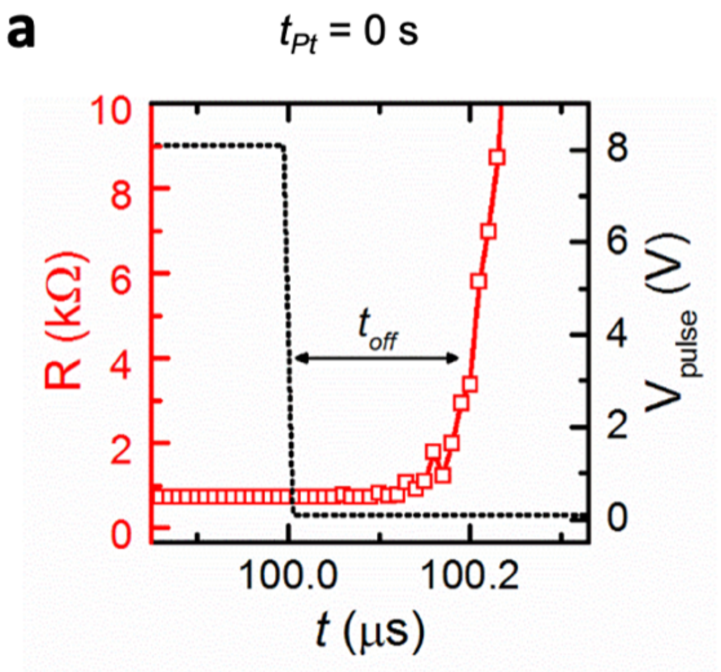


**Supplementary Figure 11 | Transient electrical resistance vs. time behavior of $VO_2$ threshold switch without Pt NP immediately after the 100 $\mu s$ voltage pulse is off at $T$ = 12 °C.** To evaluate how fast this volatile metallic state returns to the insulating state after the voltage pulse ceases, the resistance was monitored immediately after the pulse application of an 8.0 V amplitude and 100 $\mu s$ duration in both $VO_2$ threshold switches without Pt NP (i.e., $t_{Pt}$ = 0 sec, **Fig. S11**) and with Pt NP ($t_{Pt}$ = 5 sec, **Fig. 2g**) as a function of time._Regardless of Pt NP in $VO_2$ matrix, both threshold devices show a transient increase of resistance: Resistance remains close to metallic states for ~ 190 ns in both cases ($t_{off}$; black arrows) before rising abruptly, which is similar timescale (~ 200 ns) with previous reports on metallic on state-to-insulating off state transition in $VO_2$ threshold switch (*Nature* **569**, 388 (2019)); simple heating effects by Pt NPs cannot explain the enhanced memory effect.

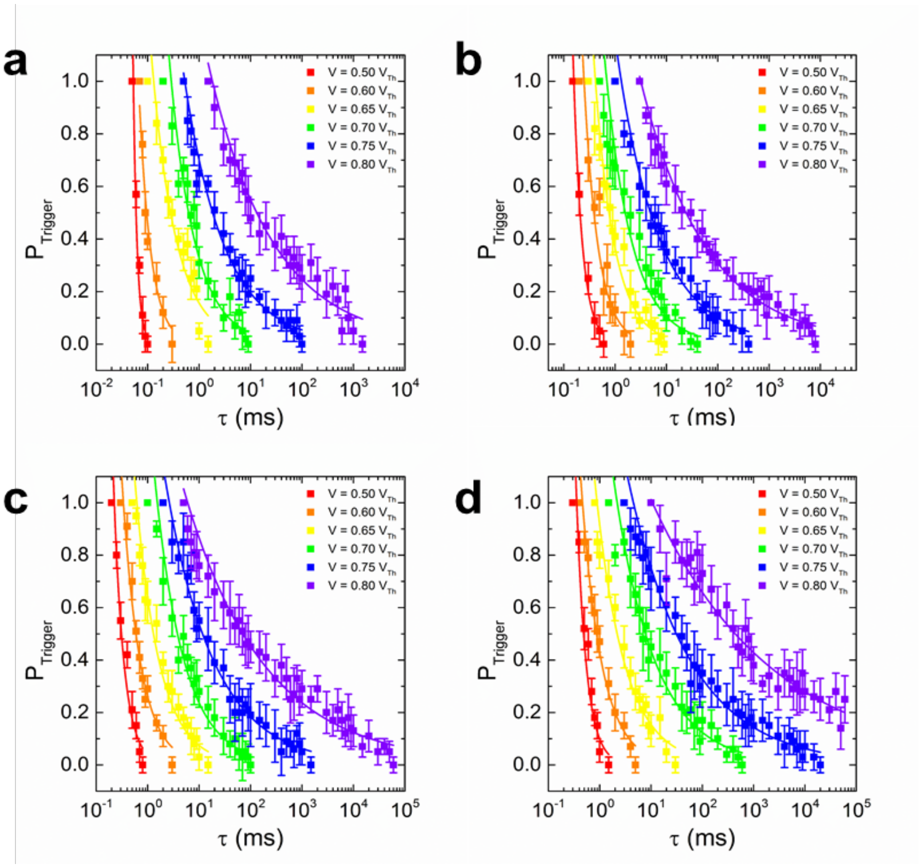


**Supplementary Figure 12 | Enhanced memory effect by subthreshold firing as a function of Pt NP coverages.** Probability that the probe pulse will trigger the insulator-to-metal transition ($P_{Trigger}$) as a function of $\tau$ at 12 °C in a $VO_2$ Mott switch with different Pt coverage (**a,** $t_{Pt}$ = 0 sec, **b,** $t_{Pt}$ = 1 sec, **c,** $t_{Pt}$ = 3 sec, **d,** $t_{Pt}$ = 5 sec). $P_{Trigger}$ is plotted for different $V_{Probe}$ amplitudes: 0.5 $V_{th,pulse}$, 0.6 $V_{th,pulse}$, 0.65 $V_{th,pulse}$, 0.7 $V_{th,pulse}$, 0.75 $V_{th,pulse}$ and 0.8 $V_{th,pulse}$. This probability was obtained after averaging 100 pump–probe protocols at each $\tau$. The error bars were calculated using the standard deviation of the binomial distribution. Between pump–probe experiments, the system was allowed to relax for several seconds to ensure complete relaxation. Solid lines are fits to curves of the type $\alpha t^{-\beta} + \gamma$, where α, β and γ are fitting parameters obtained by the least-squares method.

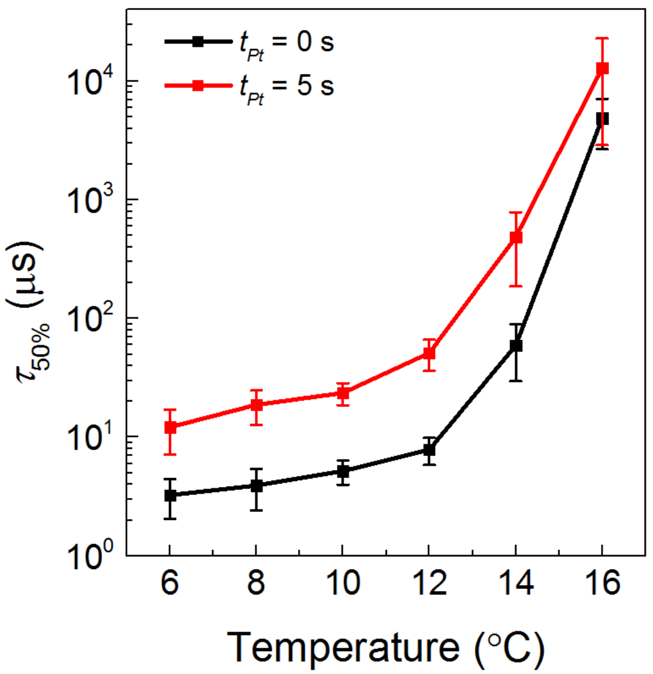


**Supplementary Figure 13 | Temperature dependence of the memory effect for two $VO_2$ devices with different Pt coverage ($t_{Pt}$ = 0 sec and $t_{Pt}$ = 5 sec).** $\tau_{50\%}$ is remarkably increased as the temperature approaches the transition temperature ($T_{IM}$ ~ 20 °C) in both cases. Interestingly, $\tau_{50\%}$ ($t_{Pt}$ = 5 sec) is up to an order of magnitude greater than $\tau_{50\%}$ ($t_{Pt}$ = 0 sec), which represent that these metastable metallic puddles are likely to "locally" remain trapped longer in Pt NP-embedded $VO_2$ films than in pure $VO_2$ films. The error bars were calculated using the standard deviation of the binomial distribution.
